# Supplementary material for: An immunostimulatory glycolipid that blocks SARS-CoV-2, RSV, and influenza infections in vivo
Source: Nat Commun. 2023 Jul 5;14:3959. doi: 10.1038/s41467-023-39738-1 (PMC10319732; doi:10.1038/s41467-023-39738-1)
Supplement: Supplementary file 1 — Supplementary Information [file 41467_2023_39738_MOESM1_ESM.pdf]

<u>**Supplementary File**</u>

An Immunostimulatory Glycolipid That Blocks SARS-CoV-2, RSV, and Influenza Infections In Vivo

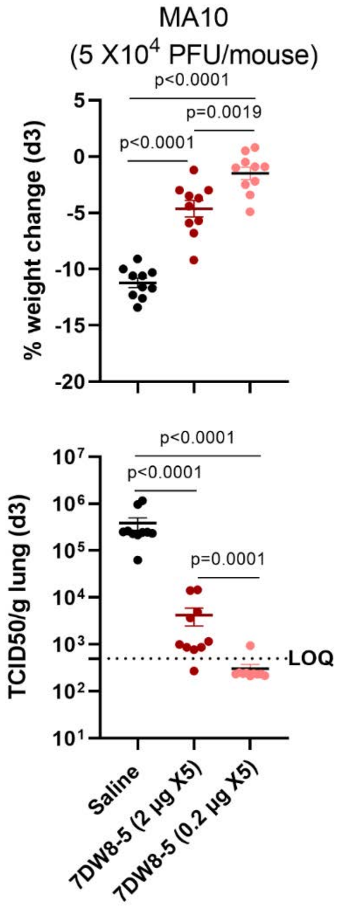

Supplementary Fig. 1. Comparison of antiviral effects after repeated intranasal dosing of a small dose (0.2 µg) and a large dose (2 µg) of 7DW8-5 every other day for 10 days. Groups of mice received 5 doses of 7DW8-5 at either 2 µg or 0.2 µg per dose prior to challenge with SARS-CoV-2 MA10 virus. Three days later, body weight changes and viral load in lungs were measured. Dotted line in the TCID50/g graph indicated the limit of quantitation of the assay. Non-parametric statistical analysis was done in GraphPad Prism v 9.3 using two-tailed Mann–Whitney *U* test, for the biological experiment (n=1). Mean ± SEM is represented for each of the above graphs.

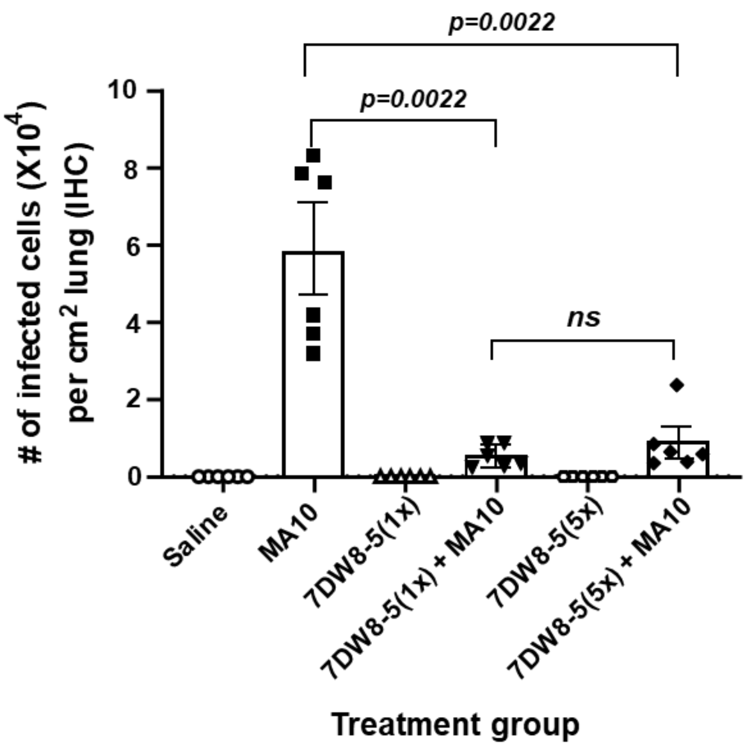

Samples	Spot number of virus-infected cells in lung IHC			
	Left	Right	Mean	SD
Slide 1 (Saline)	0	0		
Slide 2 (Saline)	0	0	0	0
Slide 3 (Saline)	0	0		
Slide 4 (MA10)	334	297		
Slide 5 (MA10)	665	254	464.5	188.2
Slide 6 (MA10)	610	627		
Slide 7 (7DW8-5 x 1)	0	0		
Slide 8 (7DW8-5 x 1)	0	0	0	0
Slide 9 (7DW8-5 x 1)	0	0		
Slide 10 (7DW8-5 x 1 + MA10)	68	23		
Slide 11 (7DW8-5 x 1 + MA10)	42	26	41.3	22.8
Slide 12 (7DW8-5 x 1 + MA10)	70	19		
Slide 13 (7DW8-5 x 5)	0	0		
Slide 14 (7DW8-5 x 5)	0	0	0	0
Slide 15 (7DW8-5 x 5)	0	0		
Slide 16 (7DW8-5 x 5 + MA10)	201	45		
Slide 17 (7DW8-5 x 5 + MA10)	30	67	70.3	65.6
Slide 18 (7DW8-5 x 5 + MA10)	51	28		

Supplementary Fig. 2. Average number of infected spots measured between each treatment group using immunostaining with SARS-CoV-2 NP antibody. The number of infected spots were counted in defined image sections of 0.8 mm^2 using Image J by setting the threshold at 0 and 145 at 16-bit. For a total of 6 images for each treatment, the number of spots counted from each IHC slide are shown in the table below. Non-parametric statistical analysis

was done in GraphPad Prism v 9.3 using Mann–Whitney *U* test (no Gaussian distribution) on the total spots counted in each treatment group. Mean ± SEM is represented for the above plot.

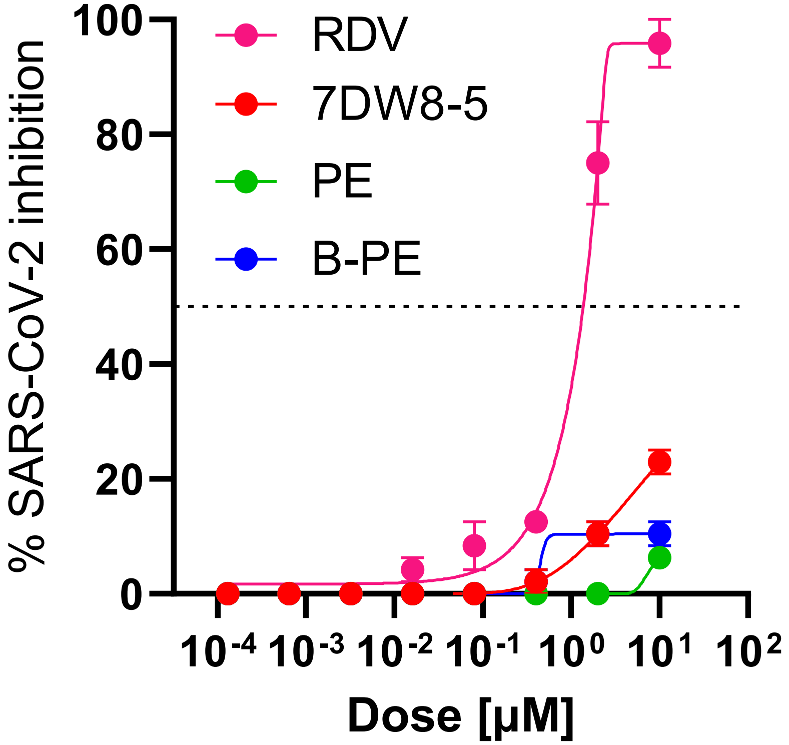

Supplementary Fig. 3. Lack of antiviral effect of 7DW8-5 on SARS-CoV-2 (USA/WA1) in VeroE6 cells as scored by reduction in cytopathic effect in vitro. Remdesivir (RDV) served as a positive control for the assay. Compounds 1,2-dioleoyl-sn-glycero-3-phosphoethanolamine-N-(hexanoylamine) (PE) and 1,2-dioleoyl-sn-glycero-3-phosphoethanolamine-N-(biotinyl) (B-PE) were included as lipid antigens known to bind to human CD1d. Mean ± SEM is represented for the above graph.

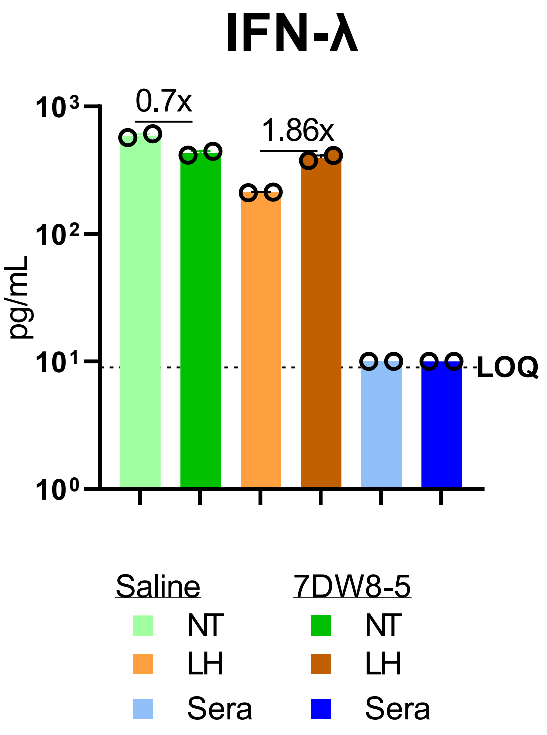

Supplementary Fig. 4. Minimal induction of IFN-λ by 7DW8-5. BALB/c mice (N=5) were given 2 μg of 7DW8-5 intranasally. Twenty-four hours later, supernatants of nasal turbinates (NT) and lung homogenates (LH) were harvested from 7DW8-5-treated and saline-treated mice. Sera were also collected. The level of IFN-λ was then analyzed by Mouse IL-28B/(IFN lambda 3) ELISA and the concentrations plotted (pg/mL). The fold increase with 7DW8-5 treatment is shown at the top of the graph. Bar graph indicates the average pg/mL for each treatment.

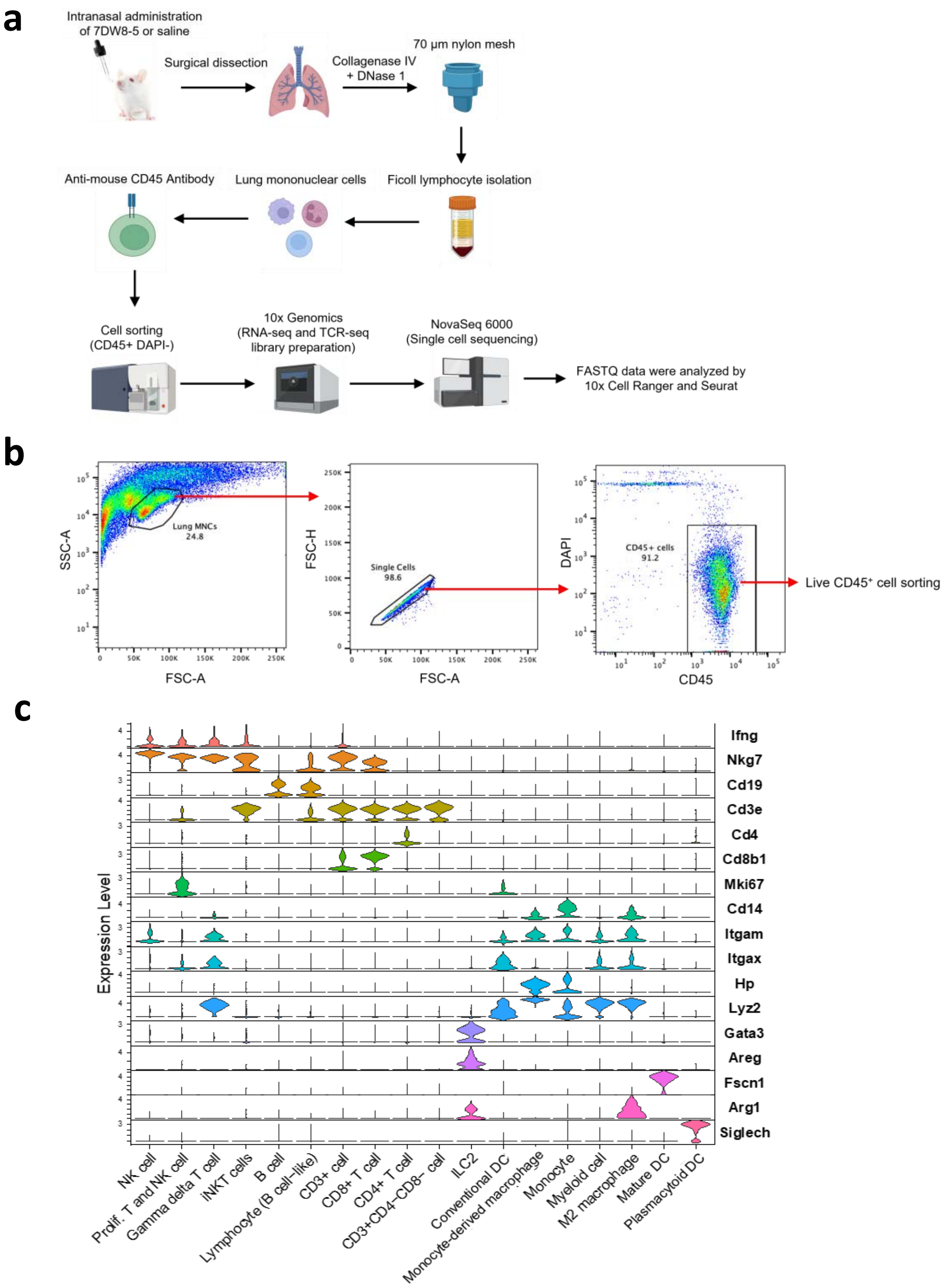

Supplementary Fig. 5. Single-cell analysis of mononuclear cells derived from the lungs of mice treated with 7DW8-5 or saline. (a) Experimental design and workflow created using purchased images from iStock photos on a template available at graphic design software, Canva (Canva.com) **(b)** Gating strategy used to sort live CD45^{+} cells. After gating mononuclear cell

(MNC) population based on their forward scatter area (FSC-A) and side scatter area (SSC-A) density plot (left panel), the single cells were gated by excluding doublets (middle panel). Finally, viable cells with DAPI negative population, which were also CD45+ cells stained with phycoerythrin-labeled anti-mouse CD45 antibody, were gated for sorting (right panel). **(c)** Profiling of marker genes across 18 distinct populations immune cells from lung homogenates of 7DW8-5-treated mice.

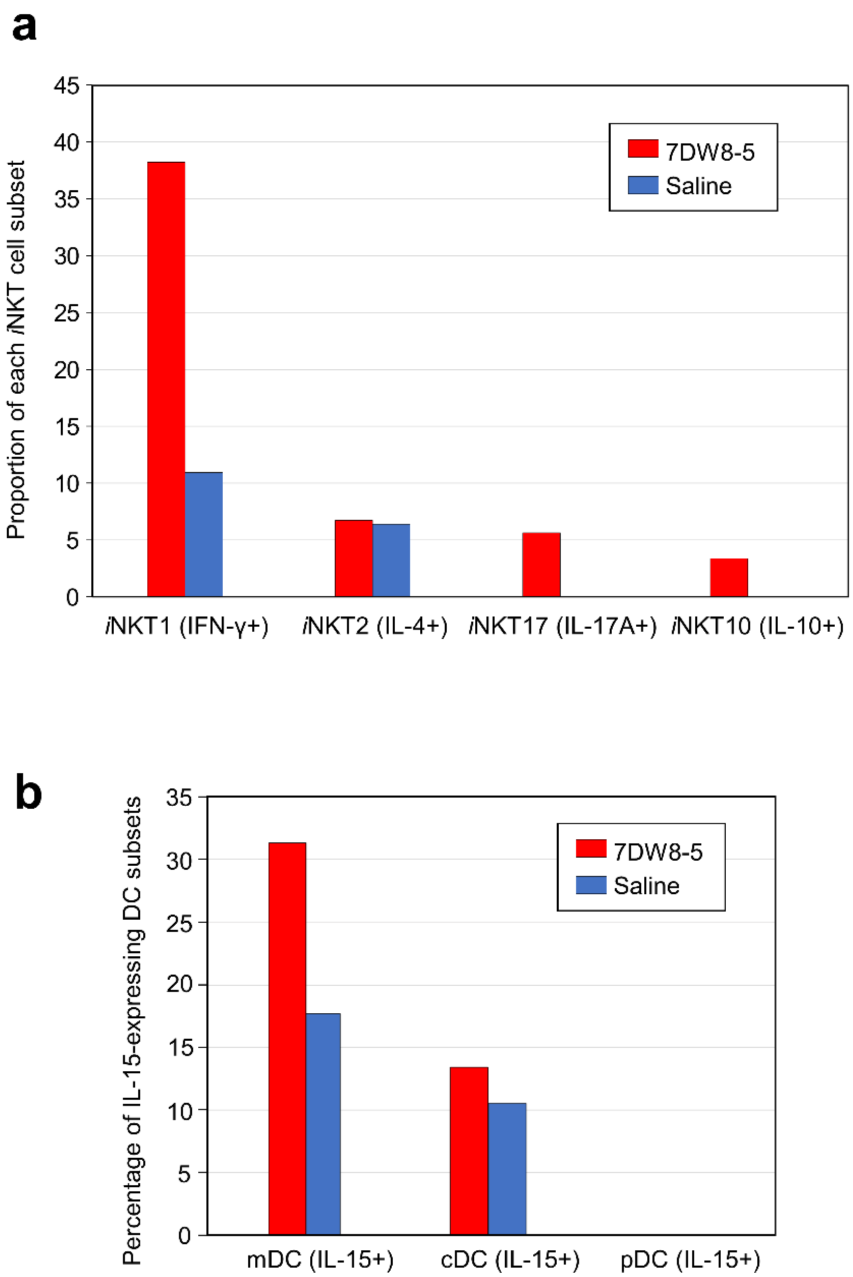

Supplementary Fig. 6. Identification of subsets of DCs and iNKT cells expanded in the lung after 7DW8-5 intranasal administration. (a) *i*NKT cell subsets in each UMAP of 7DW8-5 and saline (Fig. 3c) were categorized including iNKT1, iNKT2, iNKT17 and iNKT10 based on the expression of signature gene markers[1]. (b) Percentage of DC subsets expressing IL-15 was determined from the UMAP of 7DW8-5, comparing to UMAP of saline, shown in Fig. 3c.

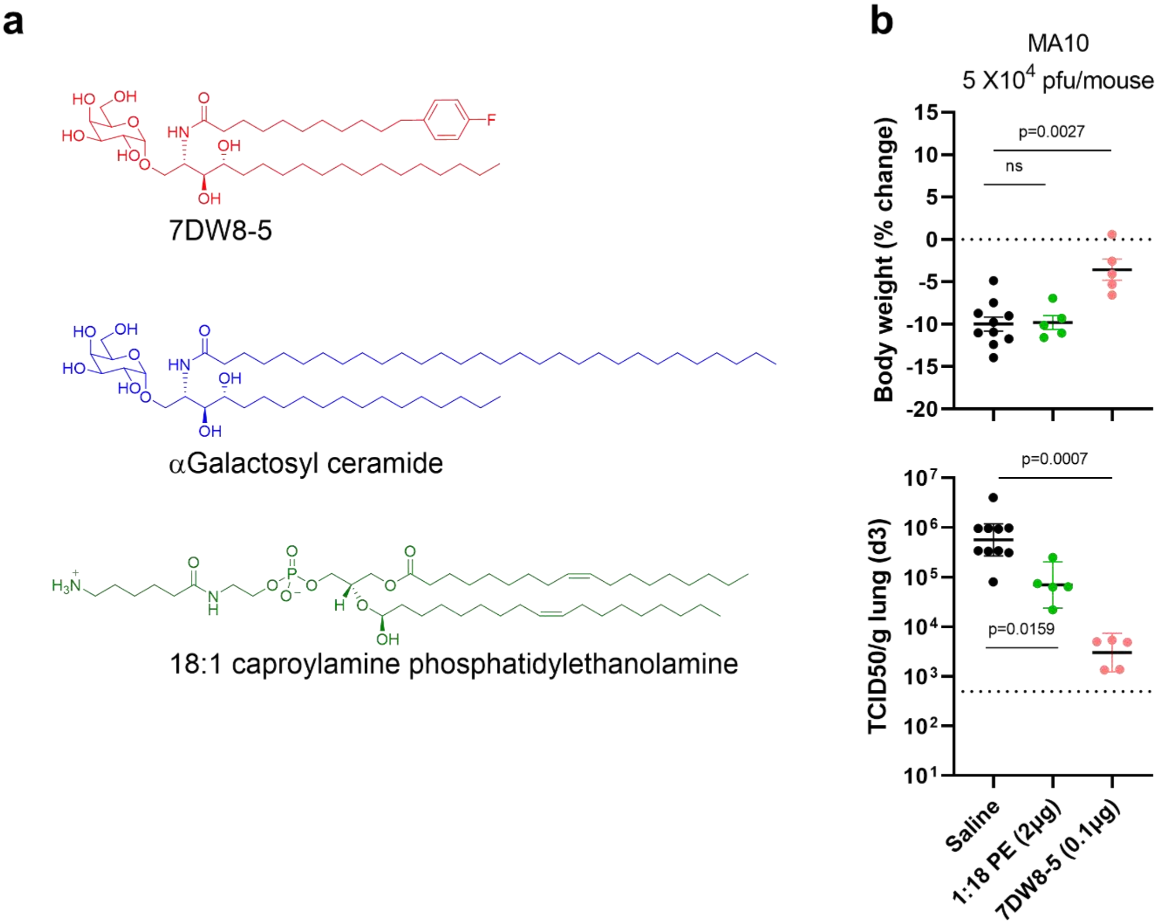

Supplementary Fig. 7. Structures and activity comparison of lipids used in this study: (a) Chemical structures of glycolipids used in the study: 7DW8-5 and its parental compound, α-galactosylceramide, along with an inactive control 18:1 caproylamine phosphatidylethanolamine (PE). **(b)** Comparison of body weight and viral load in lung homogenates of BALB/c mice dosed with 7DW8-5 (0.1 μg) versus 18:1 PE (2 μg) 24h prior to challenge with SARS-CoV-2 MA10 virus and collected 72 h post challenge. The dotted line in the TCID50/g graph indicates the limit of quantitation of the viral load assay. Non-parametric statistical analysis was done in GraphPad Prism v 9.3 using two-tailed Mann–Whitney *U* test and shown in the figure displaying one of the two similar biological experiments. Mean ± SEM is represented for the graph.

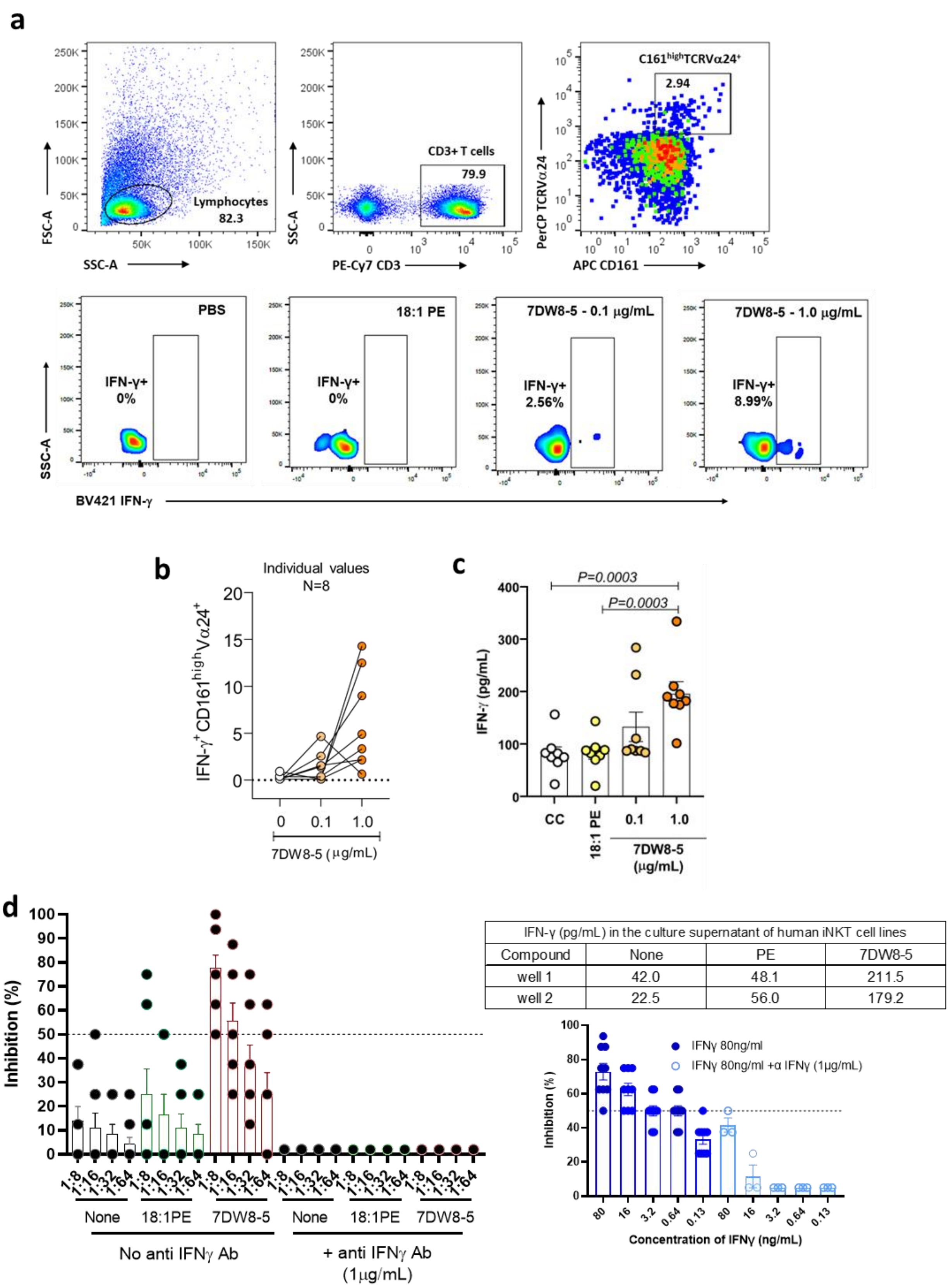

IFN-γ (pg/mL) in the culture supernatant of human iNKT cell lines			
Compound	None	PE	7DW8-5
well 1	42.0	48.1	211.5
well 2	22.5	56.0	179.2

Supplementary Fig. 8. Activation of human *i*NKT cells by 7DW8-5 *in vitro* and inhibition of SARS-CoV-2 infection of Huh7 cells by supernatant of 7DW8-5-activated human *i*NKT cells: **a.** A flow cytometric analysis shows that 7DW8-5 activates human CD161^{high}TCRVα24+ NKT cells to secrete IFN-γ *in vitro*. The gating strategy was comprised of first selecting the lymphocyte population based on density plots built by side scatter area (SSC-A) versus forward scatter area (FSC-A). Within lymphocytes, CD3+ T-cell population was further selected by identifying cells stained with PE-Cy7-labeled anti-human CD3 antibody among a low granularity lymphocyte population based on the SSC-A density plot. Then, CD161^{high}TCRVα24+ cells were selected by positive staining with both PerCP-labeled anti-TCRVα24 antibody and APC-labeled anti-human CD161 antibody. Within CD161^{high}TCRVα24+ cells, cells that express IFN-γ were identified by positive staining with BV421-labeled anti-human IFN-γ antibody. Results are expressed in percentage of positive cells. **b.** 7DW8-5 activates human CD161^{high}TCRVα24+ NKT cells from 8 individuals in a dose dependent fashion. **c.** Dose dependent IFN-γ secretion by 7DW8-5-activated human PBMCs from 8 individuals. Each point is the pg/mL of IFN-γ secreted by a single donor sample. Non-parametric statistical analysis was done in GraphPad Prism v 9.3 using two-tailed Mann–Whitney U test and shown in the figure displaying one of the two similar biological experiments. Mean ± SEM is represented for the graph. **d.** Human iNKT cell lines from 3 different donors were cocultured with Hela cells transfected with human CD1d in the presence of 1 µg/mL of 7DW8-5 or 18:1 PE or saline for 24h and the supernatants were collected and pooled. The amount of IFN-γ in the supernatants was determined by ELISA. Supernatants were then serially diluted (2-fold) from 1:8 to 1:64 dilution and incubated on a monolayer of Huh7 cells overnight. A serial 5-fold dilution of recombinant IFNγ was used as positive control. In addition, supernatants were mixed with anti-human IFNγ antibody (1µg/mL). Following this incubation, the cells were infected with ic-SARS-CoV-2-mNeon isolate and incubated further for 24h. Cells were examined under fluorescent microscope to enumerate GFP^{+} cells and percentage inhibition was calculated as the difference in fluorescence in the test wells compared to wells receiving no treatment (virus only) and plotted using GraphPad Prism v 9.3. Each point represents a replicate of the inhibition of the virus (%) by the indicated dilution of the activated supernatant following the corresponding treatment while the bar graph represents the average inhibition represented by a mean ± SEM of the points for that dilution. For the IFN-γ dilutions, similarly, the individual points are shown in the spheres while the mean ± SEM is represented by the bar graph. Non-parametric statistical analysis was done in GraphPad Prism v 9.3 using two-tailed Mann–Whitney *U* test

Supplementary References

1. Georgiev, H., Ravens, I., Benarafa, C., Forster, R. & Bernhardt, G. Distinct gene expression patterns correlate with developmental and functional traits of iNKT subsets. *Nat Commun* **7**, 13116 (2016).
