# Supplementary material for: An immunostimulatory glycolipid that blocks SARS-CoV-2, RSV, and influenza infections in vivo
Source: Nat Commun. 2023 Jul 5;14:3959. doi: 10.1038/s41467-023-39738-1 (PMC10319732; doi:10.1038/s41467-023-39738-1)
Supplement: Supplementary file 3 — Reporting Summary [file 41467_2023_39738_MOESM3_ESM.pdf]

nature portfolio

Corresponding author(s): DAVID D HO, MORIYA TSUJI, YAOXING HUANG

Last updated by author(s): Jun 21, 2023

Reporting Summary

Nature Portfolio wishes to improve the reproducibility of the work that we publish. This form provides structure for consistency and transparency in reporting. For further information on Nature Portfolio policies, see our Editorial Policies and the Editorial Policy Checklist.

Statistics

For all statistical analyses, confirm that the following items are present in the figure legend, table legend, main text, or Methods section.

n/a	Confirmed	
☐	☒	The exact sample size (*n*) for each experimental group/condition, given as a discrete number and unit of measurement
☐	☒	A statement on whether measurements were taken from distinct samples or whether the same sample was measured repeatedly
☐	☒	The statistical test(s) used AND whether they are one- or two-sided<br>*Only common tests should be described solely by name; describe more complex techniques in the Methods section.*
☐	☒	A description of all covariates tested
☐	☒	A description of any assumptions or corrections, such as tests of normality and adjustment for multiple comparisons
☐	☒	A full description of the statistical parameters including central tendency (e.g. means) or other basic estimates (e.g. regression coefficient) AND variation (e.g. standard deviation) or associated estimates of uncertainty (e.g. confidence intervals)
☐	☒	For null hypothesis testing, the test statistic (e.g. *F*, *t*, *r*) with confidence intervals, effect sizes, degrees of freedom and *P* value noted<br>*Give P values as exact values whenever suitable.*
☒	☐	For Bayesian analysis, information on the choice of priors and Markov chain Monte Carlo settings
☒	☐	For hierarchical and complex designs, identification of the appropriate level for tests and full reporting of outcomes
☒	☐	Estimates of effect sizes (e.g. Cohen's *d*, Pearson's *r*), indicating how they were calculated

Our web collection on statistics for biologists contains articles on many of the points above.

Software and code

Policy information about availability of computer code

Data collection	Cell Ranger v 6.1.2 (10x Genomics) was used to process the sequences obtained from the 5' transcript and TCR-seq libraries. SoftMax Pro 7.0.2 (Molecular Devices LLC) was used to collect the fluorescence data for neuraminidase assay for measuring influenza PR8 titers. Image J 1.38 e (NIH) was used to count the number of infected spots in the immunofluorescence sections. Illumina HiSeQ was used to sequence the labeled RNA library. 10x Chromium single-cell 5′ reagent kits v2 (Dual Index) was used to prepare the single cell RNA seq and single cell TCR seq libraries. pooled libraries were sequenced on Illumina NovaSeq 6000 system. FACS was perfromed using BD LSR Fortessa (BD Biosciences, San Jose, CA) Flow Cytometer and data acquisition was performed using the FACS DIVA software (BD Biosciences, San Jose, CA).
Data analysis	Seurat 4.1.0 package (Satija lab) was used to combine and integrate metadata for TCR clonotypes and sequences. Flow cytometry data analysis was performed using FlowJo software (version 10.6.2, BD Biosciences, San Jose, CA). GraphPad Prism v 9.3. was used for analyses of body weight and viral load data analyses, statistical measurements, graphing and figure viewing from all in vivo experiments performed. Biorender was used to create graphics for Figure 1a. iStock images were purchased to create a design and workflow of the single-cell analysis of mononuclear cells at Canva.com, a graphic design platform. Schematic showing the interaction between iNKT cells that carry iTCR and dendritic cells (DC) bearing CD1d that is bound to the glycolipid 7DW8-5 in figure 1a was created using BioRender.

For manuscripts utilizing custom algorithms or software that are central to the research but not yet described in published literature, software must be made available to editors and reviewers. We strongly encourage code deposition in a community repository (e.g. GitHub). See the Nature Portfolio guidelines for submitting code & software for further information.

Data

Policy information about availability of data

All manuscripts must include a data availability statement. This statement should provide the following information, where applicable:

- Accession codes, unique identifiers, or web links for publicly available datasets
- A description of any restrictions on data availability
- For clinical datasets or third party data, please ensure that the statement adheres to our policy

mRNA sequencing data for comparing differential gene expression and RNA-sequencing data associated with single-cell RNA seq experiments have been made available on the European bioinformatics Institute (EBI) server (https://www.ebi.ac.uk/biostudies/arrayexpress) via the ArrayExpress database using accession codes E-MTAB-12345 for mRNA and E-MTAB-11770 for the single cell sequencing. Materials used in this study will be made available but may require execution of a materials transfer agreement. All the data are provided in the paper or the Supplementary Information. Source data are provided with this paper.

Research involving human participants, their data, or biological material

Policy information about studies with human participants or human data. See also policy information about sex, gender (identity/presentation), and sexual orientation and race, ethnicity and racism.

Reporting on sex and gender	Not applicable.
Reporting on race, ethnicity, or other socially relevant groupings	Not applicable.
Population characteristics	Not applicable.
Recruitment	Not applicable.
Ethics oversight	All animal experiments were carried out in strict accordance with the Policy on Humane Care and Use of Laboratory Animals of the United States Public Health Service. The protocol was approved by the Institutional Animal Care and Use Committee (IACUC) at The Columbia University (Animal Welfare Assurance no. D16-00003) and the Washington University School of Medicine (Animal Welfare Assurance no. A3381–01).

Note that full information on the approval of the study protocol must also be provided in the manuscript.

Field-specific reporting

Please select the one below that is the best fit for your research. If you are not sure, read the appropriate sections before making your selection.

☒ Life sciences ☐ Behavioural & social sciences ☐ Ecological, evolutionary & environmental sciences

For a reference copy of the document with all sections, see nature.com/documents/nr-reporting-summary-flat.pdf

Life sciences study design

All studies must disclose on these points even when the disclosure is negative.

Sample size	Power analysis based on guidelines by Institute for Laboratory Animal Research were used to predetermine sample size to estimate minimum number of animals required to detect significant effect of a glycolipid, if one is seen.<br>In vitro assays were performed with a minimum number of doses between the tested glycolipids required to confirm i a significant difference. Experiments with N=2 or N=3 in the manuscript were therefore chosen.
Data exclusions	No data were excluded.
Replication	Virus titration experiments were performed from lung or other tissue homogenates in at least two successful replicates. Since data from two rounds were very similar, only a single round of experimentation is displayed in the manuscript using the mean and standard error between the groups. For experiment in Figure 4a, results were combined from two experiments (based on the rigor of power-law) based on repeat experiments during revisions of the manuscript, to accurately reflect the comparison of the anti-SARS-CoV-2 activity of 7DW8-5 and alpha-GalCer in BALB/c mice as requested by review.
Randomization	As this is an observational study, randomization was not relevant.
Blinding	Blinding not performed due to observational nature of the study.

Reporting for specific materials, systems and methods

We require information from authors about some types of materials, experimental systems and methods used in many studies. Here, indicate whether each material, system or method listed is relevant to your study. If you are not sure if a list item applies to your research, read the appropriate section before selecting a response.

Materials & experimental systems

n/a	Involved in the study
☐	☒ Antibodies
☐	☒ Eukaryotic cell lines
☒	☐ Palaeontology and archaeology
☐	☒ Animals and other organisms
☒	☐ Clinical data
☒	☐ Dual use research of concern
☒	☐ Plants

Methods

n/a	Involved in the study
☒	☐ ChIP-seq
☐	☒ Flow cytometry
☒	☐ MRI-based neuroimaging

Antibodies

Antibodies used	A total of 1mg (0.5mg X 2x) of anti-mouse IFNgamma antibody (XMG 1.2; BioXcell cat # BE0055; RRID AB_1107694) or equivalent amount of anti rat IgG isotype control (HRPN; BioXCell Cat# BE0088; RRID AB_1107775) was administered to mice. BioXcell cat # BE0235 clone B133.5 (RRID AB_2687717) was used at 1ug/ml in in vitro studies to measure inhibition of effects of IFN gamma in Huh7 cells. Trustain FcX anti-mouse CD16/32 antibody (Biolegend Cat # 101320; clone 93; RRID AB_1574975) and PE anti mouse CD45 antibody (Biolegend Cat # 103106; clone 30-F11; RRID AB_312971) was used to sort lung MNC. SARS-CoV-2 Nucleocapsid Protein (HL344) Rabbit mAb (Cell Signaling Cat # 26369) and HRP Horse Anti-Rabbit IgG (Vector Laboratories Cat # MP-7401) was used as detection antibody in the immunohistochemical assays performed on tissue sections.<br>Panel of anti-human monoclonal antibodies used for the selection of TCR-Valpha24 human invariant NKT cells were as follows: Anti-human CD3 PE-Cy7 (BD Biosciences Cat # 557851 RRID: AB_396896 Clone: SK7); Anti-human CD4 APC-Cy7 (BD Biosciences Cat# 557871; RRID: AB_396913; clone: RPA-T4); Anti-human CD8 BV510 (BD Biosciences Cat # 563919; RRID: AB_2722546; clone SK1); Anti-human TCRValpha24 PerCP Cy 5.5 (Biolegend Cat # 360004; RRID: AB_2562495; Clone: C15); Anti-human CD161 AF647 (Biolegend Cat # 339910; RRID: AB_1574977; clone: HP-3G10); and Anti-human IFN-gamma BV421 (BD Biosciences; Cat # 562988; RRID: AB_2737934; clone B27)
Validation	All vendors were authenticated for purchase of antibodies. Lots of antibodies from BioXcell provides purity and binding validation for their antibodies against mouse IFN-gamma and specifications of in vitro and in vivo applications of their antibodies. Biolegend antibodies are purified by affinity chromatography and provides verification of binding reactivity of their antibodies to be specific to target host species (mice) and is validated for flow cytometry usage at dilutions indicated. HRP antibody from Vector laboratories purified from horse is verified to bear a micropolymer of HRP attached to the secondary anti rabbit IgG. Antibodies used for the selection of TCR-Valpha24 human invariant NKT cells come with specific dilutions recommended by the manufacturer. In addition, each lot of every individual antibody was individually titrated using standard laboratory operating procedures on appropriate cell populations to verify the optimal titer for detection in house.

Eukaryotic cell lines

Policy information about cell lines and Sex and Gender in Research

Cell line source(s)	Vero E6, Madin-Darby canine kidney (MDCK) and Hep2 cells were obtained from ATCC. (Vero E6: Cat # CRL-1586; MDCK Cat #CCL-34 and HEp2 cells Cat # CCL-23). Huh7 cells were procured from Japanese collection of Research Bioresources (JCRB0403).
Authentication	All cell lines were purchased from authenticated vendors and confirmed for morphology by microscopy.
Mycoplasma contamination	All cells were tested to be negative for mycoplasma using MycoAlert Mycoplasma detection kit (Lonza Cat # LT07-318)
Commonly misidentified lines<br>(See ICLAC register)	Hep2 is listed on ICLA list version 9. It is a cell line that is derived via HeLa contamination but has been validated for propagation of (RSV) A2 isolate in the laboratory prior to performing titration experiments.

Animals and other research organisms

Policy information about studies involving animals; ARRIVE guidelines recommended for reporting animal research, and Sex and Gender in Research

Laboratory animals	10-15wk old BALB/c mice; 14-15 wk old C57BL/6 mice; 10-12 wk old BALB/c mice lacking CD1d and CD1d2 gene (Strain: C.129S2-CD1tm1Gru/J); 7-8 wk old C57BL/6 mice lacking IFNg (strain: B6:129S7-Ifngtm1Ts/J); and 8-9 wks old K18-ACE2 C57BL/6J mice (strain: 2B6: Cg-Tg(K18-ACE2)2Prlmn/J) were purchased from The Jackson Laboratory (Bar Harbor ME). 5-6 wk old Syrian Hamster were purchased from Charles River laboratories (Wilmington, MA). Mice were housed in groups of 4-5 per cage and hamsters were housed alone. There was active photo-period of 12h on/off light/dark cycle. Ambient animal room temperature was maintained at 72degF ± 3deg. Room humidity was 50%, and maintained within ± 10%.
Wild animals	No wild animals were used.

Reporting on sex	Female mice and male hamsters were used due to prior literature on their permissiveness to infectious agents.
Field-collected samples	No field collected samples were used.
Ethics oversight	All animal experiments were carried out in strict accordance with the Policy on Humane Care and Use of Laboratory Animals of the United States Public Health Service. The protocol was approved by the Institutional Animal Care and Use Committee (IACUC) at The Columbia University (Animal Welfare Assurance no. D16-00003) and the Washington University School of Medicine (Animal Welfare Assurance no. A3381–01). Virus inoculations were performed under anesthesia that was induced and maintained with ketamine hydrochloride and xylazine, and all efforts were made to minimize animal suffering. Mice and hamsters were euthanized with CO2, with every effort made to minimize suffering.

Note that full information on the approval of the study protocol must also be provided in the manuscript.

Flow Cytometry

Plots

Confirm that:

☒ The axis labels state the marker and fluorochrome used (e.g. CD4-FITC).

☒ The axis scales are clearly visible. Include numbers along axes only for bottom left plot of group (a 'group' is an analysis of identical markers).

☒ All plots are contour plots with outliers or pseudocolor plots.

☒ A numerical value for number of cells or percentage (with statistics) is provided.

Methodology

Sample preparation	After humane euthanasia, the mouse was placed on its back on a surgical plate. After making an incision in the neck skin near the trachea using a scalpel, open the skin to expose the salivary glands. The salivary glands were separated by using pincers to expose the trachea surrounded by sternohyoid muscle. After placing a cotton thread under the trachea using pincers, the middle of th e exposed trachea between two cartilage rings was carefully punctured with a 26 G needle. Then, the catheter, about 0.5 cm, was inserted into the trachea, followed by stabilizing the catheter by tying the trachea around the catheter using the cotton thread placed.<br>Lungs were first inflated with 0.1 mg/ml collagenase IV and DNase I for 15 min at 37oc. Single cell suspensions were prepared by mechanical dissociation of lung tissue through a 70-µm nylon mesh. Lung cells were suspended in PBS and layered on Ficoll-Paque Lymphocyte Separation (Sigma). Cells were centrifuged at room temperature for 20min at 900 x g. Mononuclear cells were harvested from the gradient interphase.
Instrument	Cell sorting was performed using FACS Aria II Cell sorter (BD Biosciences)
Software	Data was analyzed using FlowJo v 10.8.1 software
Cell population abundance	Among mononuclear cells, 80% were CD45+ cells, which were then sorted. Purity of the sorted population was analyzed in a post-sort reanalysis. Cell counting for single cell sequencing was performed using a FACS DiVa software (BD Biosciences).
Gating strategy	Gating strategy used to sort live CD45+ cells. After gating mononuclear cell (MNC) population based on their forward scatter area (FSC-A) and side scatter area (SSC-A) density plot (Supplementary fig 5b left panel), the single cells were gated by excluding doublets (Supplementary fig 5b middle panel). Finally, viable cells with DAPI negative population, which were also CD45+ cells stained with phycoerythrin-labeled anti-mouse CD45 antibody, were gated for sorting (Supplementary figure 5b right panel).

☒ Tick this box to confirm that a figure exemplifying the gating strategy is provided in the Supplementary Information.
